# Neighborhood Environment Associates with Trimethylamine-N-Oxide (TMAO) as a Cardiovascular Risk Marker

**DOI:** 10.3390/ijerph18084296

**Published:** 2021-04-18

**Authors:** Nicole Farmer, Cristhian A. Gutierrez-Huerta, Briana S. Turner, Valerie M. Mitchell, Billy S. Collins, Yvonne Baumer, Gwenyth R. Wallen, Tiffany M. Powell-Wiley

**Affiliations:** 1 Clinical Center, National Institutes of Health, Bethesda, MD 20892, USA; gwallen@cc.nih.gov; 2 Social Determinants of Obesity and Cardiovascular Risk Laboratory, National Heart, Lung, and Blood Institute, National Institutes of Health, Bethesda, MD 20892, USA; cristhian.gutierrezhuerta@nih.gov (C.A.G.-H.); brianna.turner@nih.gov (B.S.T.); valerie.mitchell@nih.gov (V.M.M.); billy.collins@nih.gov (B.S.C.); yvonne.baumer@nih.gov (Y.B.); tiffany.powell-wiley@nih.gov (T.M.P.-W.); 3 Intramural Research Program, National Institute on Minority and Health Disparities, Bethesda, MD 20892, USA

**Keywords:** neighborhood deprivation, trimethylamine-N-oxide (TMAO), cardiovascular disease, inflammation, cytokines

## Abstract

Background: Neighborhoods and the microbiome are linked to cardiovascular disease (CVD), yet investigations to identify microbiome-related factors at neighborhood levels have not been widely investigated. We sought to explore relationships between neighborhood deprivation index (NDI) and the microbial metabolite, trimethylamine-N-oxide. We hypothesized that inflammatory markers and dietary intake would be mediators of the relationship. Methods: African-American adults at risk for CVD living in the Washington, DC area were recruited to participate in a cross-sectional community-based study. US census-based neighborhood deprivation index (NDI) measures (at the census-tract level) were determined. Serum samples were analyzed for CVD risk factors, cytokines, and the microbial metabolite, trimethylamine-N-oxide (TMAO). Self-reported dietary intake based on food groups was collected. Results: Study participants (*n* = 60) were predominantly female (93.3%), with a mean (SD) age of 60.83 (+/−10.52) years. Mean (SD) NDI was −1.54 (2.94), and mean (SD) TMAO level was 4.99 (9.65) µmol/L. Adjusting for CVD risk factors and BMI, NDI was positively associated with TMAO (β = 0.31, *p* = 0.02). Using mediation analysis, the relationship between NDI and TMAO was significantly mediated by TNF-α (60.15%) and interleukin)-1 β (IL; 49.96%). When controlling for clustering within neighborhoods, the NDI-TMAO association was no longer significant (β = 5.11, *p* = 0.11). However, the association between NDI and IL-1 β (β = 0.04, *p* = 0.004) and TNF-α (β = 0.17, *p* = 0.003) remained. Neither NDI nor TMAO was significantly associated with daily dietary intake. Conclusion and Relevance: Among a small sample of African-American adults at risk for CVD, there was a significant positive relationship with NDI and TMAO mediated by inflammation. These hypothesis-generating results are initial and need to be confirmed in larger studies.

## 1. Introduction

Social determinants of health (SDOH) are factors that occur as a result of where people live (context of people’s lives), as opposed to who people are (individual factors). Certain SDOH, including economic and social disadvantage markers, exist at the neighborhood level [1,2,3,4,5,6,7]. The term neighborhood deprivation represents markers of disadvantage linked to an individual’s immediate residential environment, including both built (physical) and social characteristics [8]. Significantly, these markers of disadvantage can adversely impact chronic disease outcomes, such as cardiovascular disease (CVD) morbidity and mortality [8]. In parallel to recent attention to SDOH in the literature, there is increasing interest in the microbiome’s role in health outcomes [9,10], particularly for CVD. The neighborhood environment as an SDOH may connect to the microbiome as emerging research suggests that the host environment, and not genetics, plays a role in shaping the microbiome [11]. Specifically, a neighborhood built environment characteristics, such as increased interaction among home occupants and indoor ventilation, may help determine the presence of microbiome species [12]. Nonetheless, investigations to identify associations between neighborhood characteristics and the microbiome, and the potential for a subsequent association with CVD risk, are limited in the literature.

African-Americans represent racially categorized individuals disproportionately affected by both chronic disease and adverse neighborhood conditions as an SDOH [2,7]. Neighborhood segregation, a form of structural racism in the United States (US), is a direct antecedent to African-Americans disproportionately living in higher deprivation neighborhoods, as segregation leads to inadequate resource distribution [2,7]. Consequences of inadequate resource distribution include adverse physical and social environments that contribute to CVD development [13]. Even when accounting for individual-level socioeconomic resources, physical and social environments related to neighborhood deprivation can influence health through various mechanisms, including alterations in health behaviors (i.e., dietary quality, physical activity, sedentary time, smoking, and use of healthcare resources), exposure to toxic waste and poor air quality, and physiologic stress [1,14,15]. However, the biological mechanisms by which neighborhood deprivation can influence health outcomes require further exploration. Identification and exploration of biomarkers that associate with neighborhood conditions may provide insight into biological mechanisms. To date, biomarkers related to neighborhood deprivation include inflammatory, metabolic, vascular, and neuroendocrine markers [16,17,18,19,20,21]. Comparatively, microbiome-related biomarkers have received less attention in the literature. Of the identified biomarkers, inflammatory and neuroendocrine markers may relate to the microbiome given the relationship of these biomarkers with chronic psychosocial stress [22] and the known role of stress on the microbiome [23].

Higher deprivation neighborhoods can also influence dietary quality due to limited access to nutritious foods and more access to lower fiber content and higher energy–density foods [24]. Some higher energy-dense foods, such as red and processed meat, contain amine nutrients involved in the endogenous production of the CVD-associated dietary-microbiome metabolite, trimethylamine-N-oxide (TMAO). Specific gut microbiome species metabolize the dietary amine nutrients choline, phosphatidylcholine, and carnitine [24]. These steps produce the TMAO precursor metabolite, trimethylamine (TMA). Trimethylamine is then transported to the liver and converted to TMAO by the hepatic enzyme flavin monooxygenase isoform 3 (FMO3) [25]. Concerning CVD, in vitro studies show that TMAO affects macrophage foam cell formation [25], cholesterol uptake [26], promotes thrombosis [27] and induces the production of inflammatory cytokines linked to atherosclerosis, such as TNF-α [28]. For instance, the Multiethnic Cohort Adiposity Phenotype Study reported that plasma levels of TMAO were related to both precursor dietary nutrients and inflammatory cytokines [29]. Considering the disproportionate cardiometabolic mortality attributable to diet choices among African-Americans [30], identifying microbiome-related markers relevant to diet choices and neighborhoods could lead to developing novel, multilevel interventions for CVD risk reduction.

**Figure 1:**
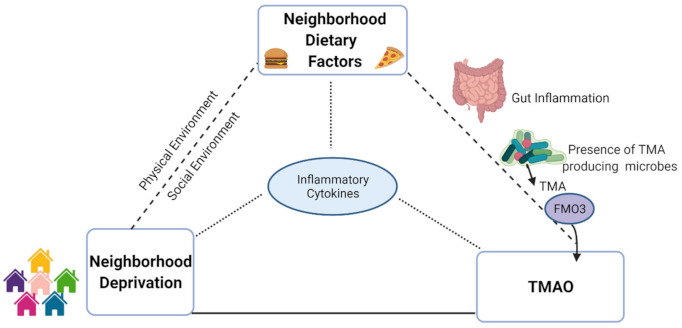
Graphical depiction of hypothesis for neighborhood-diet-inflammatory connection to explain hypothesized differences in trimethylamine-N-oxide levels based on neighborhood deprivation. NDI: neighborhood deprivation index; TMA: trimethylamine; FMO3: flavin monooxygenase isoform 3; TMAO: trimethylamine-N-oxide.

Therefore, to better understand the potential biological consequences of adverse neighborhood conditions in high-risk populations, we investigated the association between the US census tract-based neighborhood deprivation index and TMAO using cross-sectional data from African-American adults with CVD risk factors living in Washington DC-area neighborhoods. We hypothesized that neighborhood deprivation would be positively associated with TMAO levels and that potential mediators for this association would be dietary intake and markers of inflammation (Figure 1).

## 2. Materials and Methods

### 2.1. Study Design

The Washington, DC, Cardiovascular (CV) Health and Needs Assessment (DC-CHNA) was a community-based, participatory research (CBPR)-designed observational study to evaluate CV health, psychosocial factors, cultural norms, and neighborhood environment characteristics in a predominantly African-American population in Washington, DC, communities with high rates of CVD [31]. The health and needs assessment also evaluated the feasibility and acceptability of using mHealth technology in this population for objectively measuring physical activity and dietary intake [32]. To consult on the planning and implementation of the DC-CHNA, our research team partnered with a community advisory board (CAB), the DC Cardiovascular Health and Obesity Collaborative (DC CHOC), comprised of a diverse group of community leaders, church leaders, and academic partners in research [31].

### 2.2. Participant Recruitment

Partnerships between the research team and the targeted communities were facilitated by the DC CHOC and established before study recruitment and enrollment [31]. Recruitment for DC-CHNA and data collection for this study occurred between 2014 and 2018. Individuals were eligible for participation if they were between 19 and 85 years of age, members of one of the participating churches, and possessed sufficient English language proficiency to carry out study tasks. All DC-CHNA participants were enrolled in the DC CHNA clinical protocol approved by the National Heart, Lung, and Blood Institute (NHLBI) institutional review board (ClinicalTrials.gov NCT01927783).

### 2.3. Data Collection

Participants in the DC-CHNA were also recruited to the National Institutes of Health (NIH) Clinical Center for additional cardiometabolic testing and a physical examination, including clinical history and anthropometric measurements. All DC-CHNA participants (*n* = 158) were offered the opportunity to participate in this study aspect. Sixty participants accepted and came to the NIH Clinical Center, while 98 participants declined or never responded to the offer. All participants who underwent examination at the NIH Clinical Center were enrolled into a separate NHLBI Institutional Review Board-approved clinical protocol for cardiometabolic testing of individuals at risk for CVD (ClinicalTrials.gov NCT01143454). All participants signed informed consents for both the DC-CHNA and the cardiometabolic testing protocols.

### 2.4. Independent Variable: Neighborhood Deprivation Index

United States Census Bureau data from the 2010 American Community Survey was used to create a neighborhood deprivation index (NDI) for census tracts in Washington, DC and Maryland, as previously described [20]. In this study, we used census data specific to DC and Maryland to calculate the NDI. Principal axis factoring was used to identify key variables for the NDI from the following categories: income, wealth, education, employment/occupation, and housing conditions [13]. Each variable was z-standardized before factor analysis and reverse coded, as necessary. Oblique rotation was applied (minimum loading score 0.40; minimum eigenvalue 1). Cronbach’s alpha was used to measure the internal consistency of each factor (minimum alpha 0.70). Neighborhood variables that loaded into factors were log-transformed median household income, log-transformed median home value,% receiving welfare,% below the poverty level,% single mothers with children,% households without a telephone,% non-owner occupied units,% households not receiving dividends, interest, or rental income,% adults ≥ 25 years old without a high school diploma,% adults ≥ 25 years old without a Bachelor’s degree, and% working adults not in an executive, managerial, or professional occupation [8,20,33,34]. The sum of these z-standardized neighborhood variables was used to calculate a summary NDI score where a higher score represented a more deprived neighborhood. NDI calculated using this method is associated with CVD risk and has been recommended for use by expert consensus [35].

### 2.5. Dependent Variable: Trimethylamine-N-Oxide

Trimethylamine-N-oxide levels were measured from fasting serum samples obtained at the NIH Clinical Center visits. Samples were maintained at −80 °C until the samples were used for TMAO measurement. TMAO levels were measured using ELISA (BioHippo-Shanghai, China) according to the manufacturer’s recommendations.

### 2.6. Covariates

Demographic information on age, sex, race, and annual household income was self-reported by participants and obtained during physical examination during the cardiometabolic testing visit.

Body mass index (BMI) was used as a measure of obesity. To determine BMI, height was measured using a stadiometer (Perspective Enterprises, Portage, MI, USA), weight measured using a calibrated scale (Doran Scales, Inc., Batavia, IL, USA), and BMI calculated from the height and weight based on weight (kg)/height (m^2^) [36].

Dietary intake was evaluated using questions from the 2009–2010 National Health and Nutrition Examination Survey (NHANES) dietary screener questionnaire [37]. To assess vegetable and fruit consumption, participants recorded the number of times per day, per week, or per month they consumed a vegetable or fruit group (e.g., fruit juice, fruit, green leafy salad, orange-colored vegetables, other vegetables, beans). To calculate consumption (times per day), weekly frequencies were divided by seven, and monthly frequencies were divided by thirty to determine daily frequencies.

Blood samples were collected from all participants following an overnight fast. Blood samples were analyzed for basic chemistry, complete lipid profile, and high-sensitivity C-reactive protein (hs-CRP) at the NIH Clinical Center. The atherosclerotic cardiovascular disease (ASCVD) risk score was calculated for each participant based on age, race, sex, smoking status, total serum cholesterol, serum HDL cholesterol, blood pressure, diabetes status, and treatment for these conditions [38].

Proinflammatory cytokines were measured from patient serum samples maintained at −80 °C until samples were used for cytokine measurement. Interleukins (IL)-1β, IL-6, IL-8 and tumor necrosis factor (TNF)-α were measured using a multiplex ELISA (Meso Scale Diagnostics, Rockville, MD, USA), as described previously [39].

### 2.7. Statistical Analysis

Our study is a hypothesis-generating, secondary data analysis from the DC-CHNA, which was designed to provide observational data on CV health in a community-based cohort and for feasibility and acceptability testing for methods to improve CV health among community members. The DC-CHNA was not powered in terms of sample size for further hypothesis testing.

Steps involved in the statistical analyses were as follows: determination of descriptive data for independent, dependent and covariate variables, use of linear modeling to examine unadjusted (bivariate) and adjusted (multivariate) associations between NDI and TMAO, and mediation analyses.

Descriptive data for the study population used in this analysis were assessed for normality and measured as means with standard deviations for continuous variables. Unadjusted linear regression models were used to determine bivariate associations between NDI scores, TMAO levels, proinflammatory cytokines and daily dietary intake. Multivariate linear regression models were adjusted for BMI and ASCVD risk score. These variables were chosen due to the potential confounding role of CVD risk factors with TMAO and NDI. Specifically, the ASCVD risk score, which was derived in a racial/ethnically diverse population, was selected to provide a parsimonious measurement of CVD risk because of our small sample size and the study’s racial composition cohort. CVD-related variables that were included within the ASCVD risk score were not added as separate covariates in the model.

We also examined the impact of clustering of the study participants at the census tract level on relationships between NDI and the biomarkers found to be significantly associated with NDI. We used linear mixed modeling of the NDI-biomarker associations to account for the random effects of clustering at the census tract level. Linear mixed modeling adjusting for ASCVD risk score and BMI was done using proc glinmix in SAS (SAS Institute Inc 2013, Cary, NC, USA). The SAS code is available in Supplementary Figure S1 (See Figure S1).

Mediation analyses with structural equation modeling were used to determine the extent to which the association between NDI and TMAO levels was due to candidate mediation variables (i.e., proinflammatory cytokines and dietary intake measures). Specific candidate mediation variables were selected if the variables were significantly statistically related to both NDI and TMAO. The “proportion mediated” in the model was defined as the proportion of the effect size without the mediator (“total effect”) reduced when the mediator was included in the regression model (“direct effect”). Decisions and explanations for mediation analyses and model development were based on both effect sizes and *p* values [40,41]. Statistical significance level was determined by *p*-values < 0.05. STATA (StataCorp. 12. College Station, TX, USA: StataCorp LLC.) was used for all bivariate, multivariate and mediation analyses.

## 3. Results

The study participants (*n* = 60) were 93.33% women, with a mean age of 60.83 (SD 10.52) years (Table 1). The mean BMI was 33.00 (SD 7.85), consistent with the study population having obesity, on average. In terms of medical history, 21.67% had type 2 diabetes mellitus, 55% had a history of hyperlipidemia, and 63.33% had a history of hypertension. The mean ASCVD risk score was 10.75 (SD 8.51), representing an average, intermediate CVD risk within this sample population [38]. The mean TMAO level was 4.99 (9.65) µmol/L, comparable to TMAO levels reported in other studies [29,42].

The mean NDI level was −1.54 +/− 2.94. The range for NDI level was −7.43 to 10.33. In total, 48 census tracts were represented by our participants, and an estimated 47–50 neighborhoods were represented between Washington, DC and suburban DC, Maryland.

To determine bivariate relationships between NDI and TMAO with sociodemographics, CVD risk markers and inflammatory markers, unadjusted linear regression models were conducted. As shown in Table 2, there was a statistically significant association between NDI levels and TMAO serum levels (β = 0.33, *p* = 0.01). In the unadjusted models, neither NDI nor TMAO was associated with other CVD risk factors, including BMI. Both NDI and TMAO were positively associated with IL-1 β with β = 0.49, *p* < 0.001 and β = 0.35, *p* = 0.006, respectively. These were also positively associated with TNF- α with β = 0.49, *p* < 0.001 and β = 0.44, *p* < 0.001, respectively.

As shown in Table 3, after adjusting for the ASCVD risk score and BMI, NDI and TMAO remained positively associated (β = 0.31, *p* = 0.02). The association between NDI and inflammatory cytokines also remained significant after adjusting for ASCVD risk score and BMI. There was a significant association between NDI and both IL-1β (β = 0.49, *p* < 0.001) and TNF-α (β = 0.50, *p* < 0.001), after adjusting for ASCVD and BMI (Table 3). TMAO was positively associated with levels of the proinflammatory cytokines, IL-1β (β = 0.35, *p* = 0.007), TNF-α (β = 0.43, *p* = 0.001) and IL-8 (β = 0.46, *p* = <0.001). Neither NDI nor TMAO were significantly associated with IL-6 (β = 0.18, *p* = 0.17; β = −0.02, *p* = 0.89).

When controlling for ASCVD risk score, BMI, and the clustering of individuals within the same census tract, the association between NDI and TMAO was no longer significant (β = 5.11, *p* = 0.11). The relationship between NDI and cytokines remained significant for TNF- α (β = 0.17, *p* = 0.003) and IL-1 β (β = 0.04, *p* = 0.004).

**Figure 2:**
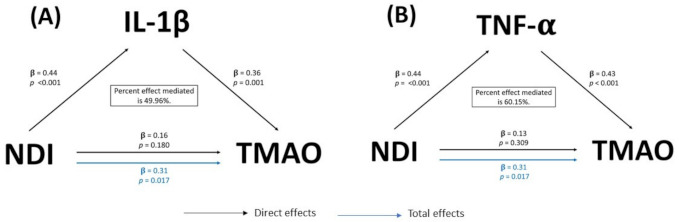
(**A**) Mediation model of the observed relationship between neighborhood deprivation index (NDI) and trimethylamine-N-oxide (TMAO) with IL-1β as a mediator, model adjusted for ASCVD risk score and BMI, (*n* = 59). (**B**) Mediation model of the observed relationship between neighborhood deprivation index (NDI) and trimethylamine-N-oxide (TMAO) with TNF-α as a mediator, model adjusted for ASCVD risk score and BMI (*n* = 59). IL-1β was undetectable in one individual (*n* = 59). ASCVD risk score includes sex, age, race, total cholesterol, HDL-C, systolic blood pressure, personal history of diabetes, personal history of smoking, personal history of treatment for hypertension. ASCVD: atherosclerotic cardiovascular disease; BMI: body mass index; IL: interleukin; TNF: tumor necrosis factor.

Mediation analysis showed that the inflammatory cytokines, IL-1β and TNF-α, mediated the relationship between NDI and TMAO. IL-1β mediated 49.96% of the relationship between NDI and TMAO (Figure 2A). In comparison, TNF-α mediated 60.15% of the NDI-TMAO relationship (Figure 2B).

Following our hypothesis, associations between NDI and dietary intake were evaluated separately as were associations between dietary intake and TMAO. There were no significant associations between NDI and mean dietary intake in adjusted models. Analysis of mean intakes for daily consumption of specific foods found no specific foods or food groups associated with TMAO after adjustment for ASCVD and BMI (Table 4). Intake of fresh, frozen, or canned fruit was positively associated with IL-6 (β = 0.34, *p* = 0.01) and TNF-α (β = 0.34, *p* = 0.02), and the intake of processed meats with IL-8 (β = 0.34, *p* = 0.02). Daily intake of vegetables, other than leafy greens and orange-colored vegetables, was positively associated with IL-1β (β = 0.33, *p* = 0.03) and IL-6 (β = 0.30, *p* = 0.04).

## 4. Discussion

In our analysis of community-dwelling African-American adults with risk factors for CVD, we found a significant positive association between NDI and TMAO levels, even when controlling for ASCVD risk factors and BMI. To our knowledge, this is one of the first studies to report a relationship between NDI, as a social determinant of health, and TMAO, a biomarker that is linked to CVD. Although other studies have assessed TMAO in multi-ethnic studies [28,41], neighborhood-level analyses were not reported in the literature. Despite our initial hypothesis, we found the association between NDI and TMAO was not related to the frequency of specific food group intake. However, the NDI-TMAO relationship was mediated by two proinflammatory cytokines, TNF-α and IL-1β, independent of ASCVD risk score and BMI. Although our findings are exploratory, they may lead to the generation of hypotheses needed for future investigations to determine specific biological mechanisms connecting neighborhood environment and TMAO.

### 4.1. Role of Inflammation in NDI and TMAO

We found that the cytokines, Il-1β and TNF-α, mediated the relationship between NDI and TMAO levels, suggesting inflammation may be a primary biologic mechanism. While associations between neighborhood deprivation and the presence of inflammatory cytokines are reported in prior studies [16,17,18,19], our study found NDI was significantly associated with the proinflammatory cytokines, Il-1 β and TNF-α. In contrast to those studies, we did not find a significant relationship with NDI and Il-6 or hsCRP. This is surprising given the literature and because our sample demonstrated considerably elevated hsCRP levels that can indicate proinflammatory processes.

Our finding that TMAO was positively associated with the proinflammatory cytokines, TNF-α and IL-1 β, is consistent with several literature studies [35,43,44]. In vitro studies by Seldin et al. [45] report TNF-α can increase endothelial cells from exposure to TMAO and that this is due to the transcription factor NF-κB. In humans, Chou et al. [46] found a positive relationship between TMAO and IL-1β among patients with stable angina. Similar to our study population of free-living adults with risk factors for CVD, Rohrmann et al. [47] found a positive association between TMAO and inflammation among German adults. Taken together, these findings support the role of TMAO in inflammatory pathways, suggesting that TMAO, at a minimum, is a marker of proinflammatory cellular processes [47,48] that are linked to atherosclerosis.

### 4.2. Role of Neighborhood Factors

The variables selected for use within our NDI calculation were related to neighborhood socioeconomic factors [20] and are associated with CVD in the literature [13]. Clustering of individuals within neighborhoods is important to consider when teasing out the independent association of NDI with biomarkers. When we accounted for clustering in the relationship between NDI and TMAO, the association was no longer statistically significant. Moreover, the association between NDI and the inflammatory markers, TNF-α and IL-β, remained significant when adjusting for clustering. Our study may be underpowered to identify an NDI-TMAO relationship when accounting for clustering. However, the connection between NDI and TMAO may be related to neighborhood location or specific intra-census tract conditions. As a result of this finding, identifying mechanisms linking neighborhood environment and TMAO could focus on neighborhood-specific factors outside of neighborhood socioeconomic factors. Candidate neighborhood factors may include food environments or localized exposure to environmental pollutants. For example, the hepatic enzyme, FMO3, needed to convert TMA into TMAO, can be activated by exposure to dioxins as environmental pollutants [49]. To date, only one study has observed and reported this environmental link, but neighborhood factors and associations were not characterized in the study. In addition, the findings related to clustering in our results could reflect the role of census tract-level factors on gut microbiota [12].

Psychosocial factors, such as chronic stress, may also cluster among individuals within a neighborhood, as well as across neighborhoods with higher deprivation [21,50,51]. Previous studies suggest that chronic stress can affect changes in the intestinal physiology that may lead to gut microbiome changes [52,53,54]. Bifidobacterium and Lachnospiraceae are two gut bacteria species that produce TMA, the TMAO precursor, and are also linked to stress exposure [55,56,57]. However, studies exploring the link between TMA-producing gut bacteria across stress and neighborhood phenotypes are not present in the current literature. Given the connection of stress with higher neighborhood deprivation [13,20], multilevel interventions around stress mitigation or perception could serve a role in mitigating neighborhood-associated factors related to the gut microbiome. These interventions could include health communication and messaging campaigns on stress awareness (institutional and societal level), identification of safe locations within a neighborhood, such as green spaces, that can mitigate stress (neighborhood level), and family-based interventions on instrumental and emotional social support (interpersonal level). These interventions are also important to consider implementing across the life course, often given life-long exposure to similar neighborhoods [58]. Mitigating the effects of stress could additionally involve novel multilevel interventions targeting microbiome diversity. Examples of possible interventions include increased affordability of foods that improve microbiome diversity, such as prebiotics, in high deprivation neighborhoods (policy level), health messaging within neighborhoods highlighting pre-biotic foods that are congruent with cultural norms (institutional and societal level), and promotion of probiotic supplements and vitamin-based interventions (individual level) shown to reduce TMA producing bacteria, such as vitamin D supplementation [59].

Diet quality is associated with NDI in the US [60] and globally [61]. Proof-of-concept feeding studies have undoubtedly shown a role for dietary intake of dietary amine precursors to increase TMAO levels through the presence of TMA-producing gut microbiome species [62]. However, studies measuring diet and nutrients within cross-sectional and longitudinal studies have not shown consistent relationships with TMAO and dietary intake [29,42,47]. Lack of consistent associations between diet and TMAO could be a function of inherent complexities of diet and endogenous TMAO production [63,64]. For example, polyphenols [65,66] and cruciferous vegetables [67] can affect TMAO production at the microbial and FMO3 enzyme levels, respectively. Our findings are consistent with studies that have not found a relationship between diet intake and TMAO. Our results may be due to the small sample size for dietary intake data or using a dietary measurement tool based on recall and reporting frequency of food groups instead of more proximal dietary measurements, such as repeated multipass 24 h dietary recall interviews [68]. Despite our sample size, we did identify a positive association between dietary intake of fruit and non-leafy green vegetables with TNF-α and IL-1β, respectively. For both fruit and other vegetables, the dietary measurement tool used in our study includes reporting fresh and processed forms. This may explain the association with the inflammatory markers and suggests that using measurement tools that specifically determine fresh versus processed food sources may be important. Lastly, we did not measure food insecurity or food environment variables in our sample. These measures of food access are important to consider when exploring the relationship between NDI and TMAO.

### 4.3. Strengths and Limitations

Although our study has significant strengths, such as using a community-based sample, using a census-tract level measurement of neighborhood deprivation and controlling for clustering within neighborhoods, our analysis has several limitations. Our study’s primary limitation is that the study design is cross-sectional with small sample size. This limitation has a significant effect on the interpretation of our findings. Due to the cross-sectional design, our use of mediation analysis was solely to identify associated factors that help generate future hypotheses and not to infer causal mechanisms. As a secondary analysis, we were not powered to address our research hypothesis. Lack of power possibly affected our ability to determine a mediator role for diet intake and may have affected our ability to detect significance between NDI and TMAO when adjusting for clustering. Due to a lower number of individual socioeconomic data responses, we could not use individual socioeconomic factors as a control variable. As a result of our study design and size limitations, more extensive studies are needed to confirm our findings, especially studies that account for clustering effects within neighborhoods. Another limitation to consider is our use of the 2010 US census tract data for NDI calculation. Although chosen to be consistent with prior studies [69] and our data collection period, future studies would utilize more recent census-tract level data.

Additionally, there are important demographic-related factors to consider when interpreting our study results. Our study population was entirely African-American and predominately women over the age of 50. Our study population’s homogeneity allows us to speculate that our findings are specific for older African-American women at risk for CVD. Due to ethnicity/ancestry-based differences in TMAO levels [42], and sex-hormone-driven differences in FMO3 activity [70], it is difficult to extrapolate our findings beyond our sample. Lastly, our analysis utilized the ASCVD risk score, which includes using racial categories. Although the risk score was determined from a multiracial sample population [38], using race is not a proxy measurement for SDOH relevant to CVD. As called for in recent publications on using racial categories in clinical risk scores, developing more precise measurements that include SDOH are needed [71,72].

## 5. Conclusions

Understanding the complex biological pathways that link neighborhood environments to CVD health outcomes is essential. It can help identify novel interventions and further explain the role of social determinants of health in chronic disease. To date, attention to these biological pathways has concentrated on inflammation, neuroendocrine and metabolic factors in the context of CVD risk. Initial studies, such as ours, can help generate hypotheses for larger studies and help direct avenues for future research to determine if TMAO, and potentially other microbial metabolites, represent patterns of exposure stemming from neighborhood environments. Undoubtedly, for populations, including many African-Americans, who are affected by discriminatory practices that lead to disproportionate placement in neighborhoods with higher neighborhood deprivation, our findings may provide insight into microbiome-related mechanisms for inflammatory-based and CVD health disparities.

## Figures and Tables

**Table 1:** Characteristics of participants from Washington, DC Cardiovascular Health and Needs Assessment (*n* = 60), who provided survey data and blood samples.

Sociodemographics	Mean (SD)/Total *n* (%)
African-American	60 (100%)
Sex, female	56 (93.33%)
Age (years)	60.83 ± 10.52
Household yearly income (USD/10k) ^^^	53.48 ± 34.20
Neighborhood deprivation index (NDI)	−1.54 ± 2.94
**CVD Risk Factors**	
Type 2 diabetes mellitus	13 (21.67%)
Hyperlipidemia	33 (55.00%)
Hypertension	38 (63.33%)
Smoking history	7 (11.67%)
BMI (kg/m^2^)	33.00 ± 7.85
LDL (mg/dL) ^a^	105.5 ± 33.02
HDL (mg/dL) ^b^	66.57 ± 20.58
TG (mg/dL) ^c^	84.97 ± 26.43
TC (mg/dL) ^d^	188.98 ± 35.20
TMAO (µmol/L)	4.99 ± 9.65
Fasting insulin (mU/mL) ^e^	16.76 ± 11.98
Fasting glucose (mg/dL) ^f^	104.73 ± 16.72
ASCVD 10 y risk score (%)	10.75 ± 8.51
HOMA-IR (%)	4.28 ± 3.21
**Inflammatory Markers**	
hs-CRP ^^^^ (mg/L) ^g^	5.70 ± 9.89
IL-1β ^^^^ (pg/mL)	0.19 ± 0.24
IL-6 (pg/mL)	1.28 ± 1.09
IL-8 (pg/mL)	32.56 ± 72.90
TNF α (pg/mL)	1.61 ± 0.99

Continuous variables expressed as mean ± standard deviation. Categorical variables displayed as total *n* (%). ^^^ Some individuals decided not to disclose this information in the presented socioeconomic survey (*n* = 46). ^^^^ hs-CRP and IL-1β were undetectable in one individual (*n* = 59). ASCVD risk score includes sex, age, race, total cholesterol, HDL-C, systolic blood pressure, personal history of diabetes, personal history of smoking, personal history of treatment for hypertension. LDL: low-density lipid cholesterol; HDL: high-density lipid cholesterol; TG: triglyceride; TC: total cholesterol; HOMA-IR: homeostatic model assessment-insulin resistance; hs-CRP: high-sensitivity C-reactive protein; IL: interleukin; TNF: tumor necrosis factor. Clinical reference ranges: ^a^ LDL optimal <100 md/dL; ^b^ HDL low <40; ^c^ TG normal <150 mg/dL; ^d^ TC normal <200 mg/dL; ^e^ insulin reference range 2.6–24.9 mU/mL; ^f^ glucose 70–99 mg/dL; ^g^ Hs-CRP low-risk <1.0, average risk 1.0–3.0, high-risk > 3.0 mg/L.

**Table 2:** Bivariate associations between neighborhood deprivation index (NDI) and trimethylamine-N-oxide (TMAO) levels with sociodemographics, CVD risk factors, and inflammatory markers (*n* = 60).

	NDI	TMAO
	β (*p*-Value)	β (*p*-Value)
Sex	0.06 (0.62)	0.09 (0.48)
Age	0.06 (0.67)	−0.14 (0.301)
Household yearly income (USD/10k) **^^^**	0.09 (0.55)	−0.173 (0.25)
History of type 2 diabetes	−0.04 (0.73)	**0.35 (0.006)**
History of hyperlipidemia	−0.09 (0.47)	−0.00 (0.98)
History of hypertension	0.16 (0.21)	0.17 (0.18)
Smoking history	−0.07 (0.57)	0.13 (0.340)
BMI	0.13 (0.33)	0.10 (0.44)
LDL	0.03 (0.84)	−0.16 (0.21)
HDL	0.22 (0.09)	0.03 (0.81)
TG	−0.25 (0.06)	−0.18 (0.18)
TC	0.12 (0.37)	−0.16 (0.22)
TMAO	**0.33 (0.01)**	-
Fasting insulin	0.00 (0.98)	−0.19 (0.147)
Fasting glucose	−0.02 (0.91)	−0.01 (0.948)
ASCVD 10 y risk score	0.07 (0.60)	0.13 (0.33)
HOMA-IR	0.01 (0.94)	−0.19 (0.159)
hsCRP ^^^^	0.04 (0.77)	0.03 (0.812)
IL-1β ^^^^	**0.49 (<0.001)**	**0.35 (0.006)**
IL-6	0.17 (0.19)	−0.02 (0.87)
IL-8	0.05 (0.72)	0.43 (0.001)
TNF-α	**0.49 (<0.001)**	**0.44 (<0.001)**

Bold indicates *p*-value < 0.05. ^^^ Some individuals decided not to disclose this information in the presented socioeconomic survey (*n* = 46). ^^^^ hs-CRP was undetectable in one individual (*n* = 59). ASCVD risk score includes sex, age, race, total cholesterol, HDL-C, systolic blood pressure, personal history of diabetes, personal history of smoking, personal history of treatment for hypertension. LDL: low-density lipid cholesterol; HDL: high-density lipid cholesterol; TG: triglyceride; TC: total cholesterol; ASCVD: atherosclerotic cardiovascular disease; HOMA-IR: homeostatic model assessment-insulin resistance; hs-CRP: high-sensitivity C-reactive protein; IL: interleukin; TNF: tumor necrosis factor.

**Table 3:** Multivariate associations between neighborhood deprivation index (NDI) and trimethylamine-N-oxide (TMAO) levels with inflammatory markers, adjusted for ASCVD 10 y risk score and BMI (*n* = 60).

	NDI	TMAO
	β (*p*-Value)	β (*p*-Value)
TMAO	**0.31 (0.02)**	-
hsCRP ^^^^	0.03 (0.79)	0.03 (0.80)
IL-1β	**0.49 (<0.001)**	**0.35 (0.007)**
IL-6	0.18 (0.17)	−0.02 (0.89)
IL-8	0.07 (0.60)	**0.46 (<0.001)**
TNF-α	**0.50 (<0.001)**	**0.43 (0.001)**

Bold indicates *p*-value < 0.05. ^^^^ hs-CRP was undetectable in one individual (*n* = 59). ASCVD risk score includes sex, age, race, total cholesterol, HDL-C, systolic blood pressure, personal history of diabetes, personal history of smoking, personal history of treatment for hypertension. ASCVD: atherosclerotic cardiovascular disease; BMI: body mass index; TG: triglyceride; hs-CRP: high-sensitivity C-reactive protein; IL: interleukin; TNF: tumor necrosis factor.

**Table 4:** Multivariate associations between daily consumption of specific foods and food groups and trimethylamine-N-oxide (TMAO) levels in participant serum, neighborhood deprivation index (NDI), and inflammatory markers, adjusted for ASCVD 10 y risk score and BMI.

Food, Food Group	Mean ± SD	Range (Min, Max)	TMAO	NDI	IL-1β	IL-6	IL-8	TNF-α
			β (*p*-Value)	β (*p*-Value)	β (*p*-Value)	β (*p*-Value)	β (*p*-Value)	β (*p*-Value)
Whole grains (*n* = 52)	1.00 ± 0.78	(0.03–4.00)	−0.08 (0.56)	−0.03 (0.81)	−0.05 (0.72)	0.01 (0.95)	0.23 (0.12)	−0.07 (0.63)
Red meat (*n* = 50) ^a^	0.32 ± 0.43	(0.00–2.00)	−0.09 (0.52)	0.09 (0.55)	0.13 (0.37)	−0.07 (0.62)	0.14 (0.33)	−0.61 (0.67)
Processed meat (*n* = 51)	0.25 ± 0.27	(0.00–1.00)	0.09 (0.51)	0.08 (0.60)	0.13 (0.36)	−0.16 (0.25)	**0.34** **(0.02)**	0.26 (0.07)
Fried foods (*n* = 52) ^b^	0.28 ± 0.28	(0.03–1.00)	−0.15 (0.32)	0.21 (0.15)	0.14 (0.35)	−0.15 (0.29)	0.05 (0.75)	−0.13 (0.39)
Fast foods (*n* = 51) ^c^	0.13 ± 0.23	(0.00–1.00)	−0.09 (0.51)	0.18 (0.18)	0.001 (0.99)	−0.07 (0.60)	0.06 (0.67)	0.01 (0.97)
Regular soda (*n* = 49)	0.18 ± 0.36	(0.00–2.00)	−0.10 (0.47)	0.14 (0.32)	0.18 (0.19)	−0.20 (0.88)	0.13 (0.33)	−0.17 (0.22)
Sweetened fruit drinks (*n* = 52) ^d^	0.46 ± 1.06	(0.00–7.00)	0.13 (0.37)	0.05 (0.71)	0.06 (0.66)	−0.06 (0.67)	−0.01 (0.94)	−0.04 (0.78)
Fruit (*n* = 54) ^e^	0.80 ± 0.80	(0.00–3.00)	−0.11 (0.44)	0.09 (0.54)	0.02 (0.89)	**0.34** **(0.01)**	0.12 (0.36)	**0.34** **(0.02)**
Green, leafy salads (*n* = 49)	1.00 ± 1.47	(0.00–7.00)	−0.14 (0.35)	−0.05 (0.76)	0.13 (0.36)	0.18 (0.21)	0.01 (0.96)	0.08 (0.59)
Vegetables (*n* = 52) ^f^	0.57 ± 0.72	(0.00–4.00)	−0.04 (0.77)	0.15 (0.30)	0.10 (0.48)	0.03 (0.83)	0.09 (0.57)	0.08 (0.59)
Other vegetables (*n* = 52) ^g^	0.62 ± 0.62	(0.00–3.00)	−0.13 (0.38)	−0.05 (0.75)	**0.33** **(0.03)**	**0.30** **(0.04)**	−0.14 (0.37)	0.07 (0.66)
Beans (*n* = 52)	0.21 ± 0.26	(0.00–1.00)	−0.07 (0.63)	0.09 (0.55)	0.01 (0.96)	0.08 (0.59)	0.06 (0.70)	0.08 (0.60)
Coffee (*n* = 48)	0.60 ± 0.64	(0.00–2.00)	−0.07 (0.66)	0.06 (0.71)	0.14 (0.35)	0.01 (0.95)	−0.02 (0.91)	0.15 (0.33)

Bold indicates *p*-value < 0.05. ASCVD: atherosclerotic cardiovascular disease; BMI: body mass index; IL: interleukin; TNF: tumor necrosis factor. ^a^ Red meat: beef, pork, ham, or sausage, answers do not include chicken, turkey or seafood. ^b^ Fried foods: chips, french fries, fried meats, fried appetizers, and fried pastries. ^c^ Fast foods: breakfast, lunch, or dinner in a fast-food restaurant. ^d^ Sweetened fruit drinks: sports or energy drinks, fruit juices made at home with added sugar; does not include diet drinks or artificially sweetened drinks. ^e^ Fruit: includes fresh, frozen, or canned fruit. ^f^ Vegetables: orange-colored vegetables (i.e., sweet potatoes, pumpkin, winter squash, or carrots). ^g^ Other vegetables: not, including green leafy or lettuce salads, orange-colored vegetables, or beans.

## Data Availability

The data presented in this study may be available upon request and determination made by the corresponding and senior authors. Data are not publically available due to restrictions on sharing data in accordance with the consent provided by participants.

## Supplementary Materials

The following are available online at https://www.mdpi.com/article/10.3390/ijerph18084296/s1, Figure S1: SAS code for neighborhood clustering linear mixed model.

## References

[1] Diez Roux A.V., Mujahid M.S., Hirsch J.A., Moore K., Moore L.V. (2016). The Impact of Neighborhoods on CV Risk. Glob Heart.

[2] Barber S., Hickson D.A., Wang X., Sims M., Nelson C., Diez-Roux A.V. (2016). Neighborhood Disadvantage, Poor Social Conditions, and Cardiovascular Disease Incidence Among African American Adults in the Jackson Heart Study. Am. J. Public Health.

[3] Wing J.J., August E., Adar S.D., Dannenberg A.L., Hajat A., Sánchez B.N., Stein J.H., Tattersall M.C., Roux A.V.D. (2016). Change in Neighborhood Characteristics and Change in Coronary Artery Calcium: A Longitudinal Investigation in the MESA (Multi-Ethnic Study of Atherosclerosis) Cohort. Circulation.

[4] Kershaw K.N., Osypuk T.L., Do D.P., De Chavez P.J., Diez Roux A.V. (2015). Neighborhood-level racial/ethnic residential segregation and incident cardiovascular disease: The multi-ethnic study of atherosclerosis. Circulation.

[5] Palmer R.C., Ismond D., Rodriquez E.J., Kaufman J.S. (2019). Social Determinants of Health: Future Directions for Health Disparities Research. Am. J. Public Health.

[6] Havranek E.P., Mujahid M.S., Barr D.A., Blair I.V., Cohen M.S., Cruz-Flores S., Davey-Smith G., Dennison-Himmelfarb C.R., Lauer M.S., Lockwood D.W. (2015). Social Determinants of Risk and Outcomes for Cardiovascular Disease: A Scientific Statement from the American Heart Association. Circulation.

[7] Williams D.R., Collins C. (2001). Racial residential segregation: A fundamental cause of racial disparities in health. Public Health Rep..

[8] Diez Roux A.V. (2004). Estimating neighborhood health effects: The challenges of causal inference in a complex world. Soc. Sci. Med..

[9] Hills R.D., Pontefract B.A., Mishcon H.R., Black C.A., Sutton S.C., Theberge C.R. (2019). Gut Microbiome: Profound Implications for Diet and Disease. Nutrients.

[10] Gilbert J.A., Blaser M.J., Caporaso J.G., Jansson J.K., Lynch S.V., Knight R. (2018). Current understanding of the human microbiome. Nat. Med..

[11] Byrd D.A., Carson T.L., Williams F., Vogtmann E. (2020). Elucidating the role of the gastrointestinal microbiota in racial and ethnic health disparities. Genome Biol..

[12] Stamper C.E., Hoisington A.J., Gomez O.M., Halweg-Edwards A.L., Smith D.G., Bates K.L., Kinney K.A., Postolache T.T., Brenner L.A., Rook G.A. (2016). The Microbiome of the Built Environment and Human Behavior: Implications for Emotional Health and Well-Being in Postmodern Western Societies. Int. Rev. Neurobiol..

[13] Diez Roux A.V., Mair C. (2010). Neighborhoods and health. Ann. N. Y. Acad. Sci..

[14] Cubbin C., Winkleby M.A. (2005). Protective and harmful effects of neighborhood-level deprivation on individual-level health knowledge, behavior changes, and risk of coronary heart disease. Am. J. Epidemiol..

[15] Ribeiro A.I., Fraga S., Kelly-Irving M., Delpierre C., Stringhini S., Kivimaki M., Joost S., Guessous I., Gandini M., Vineis P. (2019). Neighbourhood socioeconomic deprivation and allostatic load: A multi-cohort study. Sci. Rep..

[16] Keita A.D., Judd S.E., Howard V.J., Carson A.P., Ard J.D., Fernandez J.R. (2014). Associations of neighborhood area level deprivation with the metabolic syndrome and inflammation among middle- and older- age adults. BMC Public Health.

[17] Hong S., Nelesen R.A., Krohn P.L., Mills P.J., Dimsdale J.E. (2006). The Association of Social Status and Blood Pressure with Markers of Vascular Inflammation. Psychosom. Med..

[18] Petersen K.L., Marsland A.L., Flory J., Votruba-Drzal E., Muldoon M.F., Manuck S.B. (2008). Community socioeconomic status is associated with circulating interleukin-6 and C-reactive protein. Psychosom. Med..

[19] Holmes L.M., Marcelli E.A. (2012). Neighborhoods and systemic inflammation: High CRP among legal and unauthorized Brazilian migrants. Health Place.

[20] Powell-Wiley T.M., Gebreab S.Y., Claudel S.E., Ayers C., Andrews M.R., Adu-Brimpong J., Berrigan D., Davis S.K. (2020). The relationship between neighborhood socioeconomic deprivation and telomere length: The 1999–2002 National Health and Nutrition Examination Survey. SSM Popul. Health.

[21] Smith J.A., Zhao W., Wang X., Ratliff S.M., Mukherjee B., Kardia S.L.R., Liu Y., Roux A.V.D., Needham B.L. (2017). Neighborhood characteristics influence DNA methylation of genes involved in stress response and inflammation: The Multi-Ethnic Study of Atherosclerosis. Epigenetics.

[22] Elliott M. (2000). The stress process in neighborhood context. Health Place.

[23] Bailey M.T., Dowd S.E., Galley J.D., Hufnagle A.R., Allen R.G., Lyte M. (2011). Exposure to a social stressor alters the structure of the intestinal microbiota: Implications for stressor-induced immunomodulation. Brain Behav. Immun..

[24] Larson N.I., Story M.T., Nelson M.C. (2009). Neighborhood environments: Disparities in access to healthy foods in the U.S. Am. J. Prev. Med..

[25] Zhu W., Gregory J.C., Org E., Buffa J.A., Gupta N., Wang Z., Li L., Fu X., Wu Y., Mehrabian M. (2016). Gut Microbial Metabolite TMAO Enhances Platelet Hyperreactivity and Thrombosis Risk. Cell.

[26] Canyelles M., Tondo M., Cedó L., Farràs M., Escolà-Gil J.C., Blanco-Vaca F. (2018). Trimethylamine N-Oxide: A Link among Diet, Gut Microbiota, Gene Regulation of Liver and Intestine Cholesterol Homeostasis and HDL Function. Int. J. Mol. Sci..

[27] Tang W.H., Wang Z., Levison B.S., Koeth R.A., Britt E.B., Fu X., Wu Y., Hazen S.L. (2013). Intestinal microbial metabolism of phosphatidylcholine and cardiovascular risk. N. Engl. J. Med..

[28] Yang S., Li X., Yang F., Zhao R., Pan X., Liang J., Tian L., Li X., Liu L., Xing Y. (2019). Gut Microbiota-Dependent Marker TMAO in Promoting Cardiovascular Disease: Inflammation Mechanism, Clinical Prognostic, and Potential as a Therapeutic Target. Front. Pharmacol..

[29] Fu B.C., Hullar M.A.J., Randolph T.W., Franke A.A., Monroe K.R., Cheng I., Wilkens L.R., Shepherd J.A., Madeleine M.M., Le Marchand L. (2020). Associations of plasma trimethylamine N-oxide, choline, carnitine, and betaine with inflammatory and cardiometabolic risk biomarkers and the fecal microbiome in the Multiethnic Cohort Adiposity Phenotype Study. Am. J. Clin. Nutr..

[30] Micha R., Peñalvo J.L., Cudhea F., Imamura F., Rehm C.D., Mozaffarian D. (2017). Association Between Dietary Factors and Mortality From Heart Disease, Stroke, and Type 2 Diabetes in the United States. JAMA.

[31] Yingling L.R., Mitchell V., Ayers C.R., Peters-Lawrence M., Wallen G.R., Brooks A.T., Troendle J.F., Adu-Brimpong J., Thomas S., Henry J. (2017). Adherence with physical activity monitoring wearable devices in a community-based population: Observations from the Washington, D.C.; Cardiovascular Health and Needs Assessment. Transl. Behav. Med..

[32] Fowler L.A., Yingling L.R., Brooks A.T., Wallen G.R., Peters-Lawrence M., McClurkin M., Wiley K.L., Mitchell V.M., Johnson T.D., Curry K.E. (2018). Digital Food Records in Community-Based Interventions: Mixed-Methods Pilot Study. JMIR Mhealth Uhealth.

[33] Andrews M.R., Tamura K., Claudel S.E., Xu S., Ceasar J.N., Collins B.S., Langerman S., Mitchell V.M., Baumer Y., Powell-Wiley T.M. (2020). Geospatial Analysis of Neighborhood Deprivation Index (NDI) for the United States by County. J. Maps.

[34] Lian M., Struthers J., Liu Y. (2016). Statistical Assessment of Neighborhood Socioeconomic Deprivation Environment in Spatial Epidemiologic Studies. Open J. Stat..

[35] Saelens B.E., Glanz K., Frank L.D., Couch S.C., Zhou C., Colburn T., Sallis J.F. (2018). Two-Year Changes in Child Weight Status, Diet, and Activity by Neighborhood Nutrition and Physical Activity Environment. Obesity.

[36] Fryer C.D., Ervin R.B. (2013). Caloric intake from fast food among adults: United States, 2007–2010. NCHS Data Brief..

[37] Ogden C.L., Carroll M.D., Kit B.K., Flegal K.M. (2012). Prevalence of obesity and trends in body mass index among US children and adolescents, 1999–2010. JAMA.

[38] Goff D.C., Lloyd-Jones D.M., Bennett G., Coady S., D’Agostino R.B., Gibbons R., Greenland P., Lackland D.T., Levy D., O’Donnell C.J. (2014). 2013 ACC/AHA guideline on the assessment of cardiovascular risk: A report of the American College of Cardiology/American Heart Association Task Force on Practice Guidelines. Circulation.

[39] Bastarache J.A., Koyama T., Wickersham N.E., Ware L.B. (2014). Validation of a multiplex electrochemiluminescent immunoassay platform in human and mouse samples. J. Immunol. Methods.

[40] Mora S., Cook N., Buring J.E., Ridker P.M., Lee I.M. (2007). Physical activity and reduced risk of cardiovascular events: Potential mediating mechanisms. Circulation.

[41] Zhao D., Post W.S., Blasco-Colmenares E., Cheng A., Zhang Y., Deo R., Pastor-Barriuso R., Michos E.D., Sotoodehnia N., Guallar E. (2019). Racial Differences in Sudden Cardiac Death. Circulation.

[42] Meyer K.A., Benton T.Z., Bennett B.J., Jacobs D.R., Lloyd-Jones D.M., Gross M.D., Carr J.J., Gordon-Larsen P., Zeisel S.H. (2016). Microbiota-Dependent Metabolite Trimethylamine N-Oxide and Coronary Artery Calcium in the Coronary Artery Risk Development in Young Adults Study (CARDIA). J. Am. Heart Assoc..

[43] Chen M.L., Zhu X.H., Ran L., Lang H.D., Yi L., Mi M.T. (2017). Trimethylamine-N-Oxide Induces Vascular Inflammation by Activating the NLRP3 Inflammasome Through the SIRT3;mtROS Signaling Pathway. J. Am. Heart Assoc..

[44] Boutagy N.E., Neilson A.P., Osterberg K.L., Smithson A.T., Englund T.R., Davy B.M., Hulver M.W., Davy K.P. (2015). Short-term high-fat diet increases postprandial trimethylamine-N-oxide in humans. Nutr. Res..

[45] Seldin M.M., Meng Y., Qi H., Zhu W., Wang Z., Hazen Z.L., Lusis A.J., Shih D.M. (2016). Trimethylamine N-Oxide Promotes Vascular Inflammation Through Signaling of Mitogen-Activated Protein Kinase and Nuclear Factor-κB. J. Am. Heart Assoc..

[46] Chou R.H., Chen C.Y., Chen I.C., Huang H.-L., Lu Y.-W., Kuo C.-S., Chang C.-C., Huang P.-H., Chen J.-W., Lin S.-J. (2019). Trimethylamine N-Oxide, Circulating Endothelial Progenitor Cells, and Endothelial Function in Patients with Stable Angina. Sci. Rep..

[47] Rohrmann S., Linseisen J., Allenspach M., von Eckardstein A., Müller D. (2016). Plasma Concentrations of Trimethylamine-N-oxide Are Directly Associated with Dairy Food Consumption and Low-Grade Inflammation in a German Adult Population. J. Nutr..

[48] Din A.U., Hassan A., Zhu Y., Yin T., Gregersen H., Wang G. (2019). Amelioration of TMAO through probiotics and its potential role in atherosclerosis. Appl. Microbiol. Biotechnol..

[49] Petriello M.C., Charnigo R., Sunkara M., Soman S., Pavuk M., Birnbaum L., Morris A.J., Hennig B. (2018). Relationship between serum trimethylamine N-oxide and exposure to dioxin-like pollutants. Environ. Res..

[50] Cutrona C.E., Wallace G., Wesner K.A. (2006). Neighborhood Characteristics and Depression: An Examination of Stress Processes. Curr. Dir. Psychol. Sci..

[51] Hackman D.A., Robert S.A., Grübel J., Weibel R.P., Anagnostou E., Hölscher C., Schinazi V.R. (2019). Neighborhood environments influence emotion and physiological reactivity. Sci. Rep..

[52] Karl J.P., Hatch A.M., Arcidiacono S.M., Pearce S.C., Pantoja-Feliciano I.G., Doherty L.A., Soares J.W. (2018). Effects of Psychological, Environmental and Physical Stressors on the Gut Microbiota. Front. Microbiol..

[53] Konturek P.C., Brzozowski T., Konturek S.J. (2011). Stress and the gut: Pathophysiology, clinical consequences, diagnostic approach and treatment options. J. Physiol. Pharmacol..

[54] Galley J.D., Mackos A.R., Varaljay V.A., Bailey M.T. (2017). Stressor exposure has prolonged effects on colonic microbial community structure in Citrobacter rodentium-challenged mice. Sci. Rep..

[55] Chen J., Chia N., Kalari K.R., Yao J.Z., Novotna M., Soldan M.M.P., Luckey D.H., Marietta E.V., Jeraldo P.R., Chen X. (2016). Multiple sclerosis patients have a distinct gut microbiota compared to healthy controls. Sci. Rep..

[56] Yang C., Fujita Y., Ren Q., Ma M., Dong C., Hashimoto K. (2017). Bifidobacterium in the gut microbiota confer resilience to chronic social defeat stress in mice. Sci. Rep..

[57] Zhang J., Song L., Wang Y., Liu C., Zhang L., Zhu S., Liu S., Duan L. (2019). Beneficial effect of butyrate-producing Lachnospiraceae on stress-induced visceral hypersensitivity in rats. J. Gastroenterol. Hepatol..

[58] Jivraj S., Murray E.T., Norman P., Nicholas O. (2020). The impact of life course exposures to neighbourhood deprivation on health and well-being: A review of the long-term neighbourhood effects literature. Eur. J. Public Health.

[59] Wang X., Li X., Dong Y. (2020). Vitamin D Decreases Plasma Trimethylamine-N-oxide Level in Mice by Regulating Gut Microbiota. Biomed. Res. Int..

[60] Conrey S., Cline A., Brokamp C., Santanello K., Piasecki A., Staat M., Payne D., Morrow A. (2020). Neighborhood Deprivation Predicts Diet Quality at One Year of Age. Curr. Dev. Nutr..

[61] Kurotani K., Honjo K., Nakaya T., Ikeda A., Mizoue T., Sawada N., Tsugane S. (2019). Japan Public Health Center-Based Prospective Study Group. Diet Quality Affects the Association between Census-Based Neighborhood Deprivation and All-Cause Mortality in Japanese Men and Women: The Japan Public Health Center-Based Prospective Study. Nutrients.

[62] Wang Z., Klipfell E., Bennett B.J., Koeth R.A., Levison B.S., Dugar B., Feldstein A.E., Britt E.B., Fu X., Chung Y.-M. (2011). Gut flora metabolism of phosphatidylcholine promotes cardiovascular disease. Nature.

[63] Manor O., Zubair N., Conomos M.P., Xu X., Rohwer J.E., Krafft C.E., Lovejoy J.C., Magis A.T. (2018). A Multi-omic Association Study of Trimethylamine N-Oxide. Cell Rep..

[64] Zhu C., Sawrey-Kubicek L., Bardagjy A.S., Houts H., Tang X., Sacchi R., Randolph J.M., Steinberg F.M., Zivkovic A.M. (2020). Whole egg consumption increases plasma choline and betaine without affecting TMAO levels or gut microbiome in overweight postmenopausal women. Nutr. Res..

[65] Chen M.L., Yi L., Zhang Y., Zhou X., Ran L., Yang J., Zhu J.-d., Zhang Q.-y., Mi M.-t. (2016). Resveratrol Attenuates Trimethylamine-N-Oxide (TMAO)-Induced Atherosclerosis by Regulating TMAO Synthesis and Bile Acid Metabolism via Remodeling of the Gut Microbiota. mBio.

[66] Barabási A.-L., Menichetti G., Loscalzo J. (2020). The unmapped chemical complexity of our diet. Nat. Food.

[67] Chen S., Henderson A., Petriello M.C., Romano K.A., Gearing M., Miao J., Schell M., Sandoval-Espinola W.J., Tao J., Sha B. (2019). Trimethylamine N-Oxide Binds and Activates PERK to Promote Metabolic Dysfunction. Cell Metab..

[68] Ahluwalia N., Dwyer J., Terry A., Moshfegh A., Johnson C. (2016). Update on NHANES Dietary Data: Focus on Collection, Release, Analytical Considerations, and Uses to Inform Public Policy. Adv. Nutr..

[69] Powell-Wiley T.M., Dey A.K., Rivers J.P., Chaturvedi A., Andrews M.R., Ceasar J.N., Claudel S.E., Mitchell V.M., Ayers C., Tamura K. (2021). Chronic Stress-Related Neural Activity Associates With Subclinical Cardiovascular Disease in a Community-Based Cohort: Data from the Washington, D.C. Cardiovascular Health and Needs Assessment. Front. Cardiovasc. Med..

[70] Razavi A.C., Potts K.S., Kelly T.N., Bazzano L.A. (2019). Sex, gut microbiome, and cardiovascular disease risk. Biol. Sex Differ..

[71] Powell-Wiley T.M. (2020). Disentangling Ancestry From Social Determinants of Health in Hypertension Disparities—An Important Step Forward. JAMA Cardiol..

[72] Yancy C.W., McNally E. (2020). Reporting Genetic Markers and the Social Determinants of Health in Clinical Cardiovascular Research—It Is Time to Recalibrate the Use of Race. JAMA Cardiol..

