# Supplementary material for: Neighborhood Environment Associates with Trimethylamine-N-Oxide (TMAO) as a Cardiovascular Risk Marker
Source: Int J Environ Res Public Health. 2021 Apr 18;18(8):4296. doi: 10.3390/ijerph18084296 (PMC8072883; doi:10.3390/ijerph18084296)
Supplement: Supplementary file 1 [file ijerph-18-04296-s001.pdf]

Supplemental Figure 1. SAS Code for Neighborhood Clustering Linear Mixed Model using separate the dependent variables: trimethylamine-N-oxide (TMAO), IL(Interleukin)-1 β, and Tumor Necrosis Factor (TNF)-α.

```
proc glimmix data=g.wgeoid;
      class geoid;
      model TMAO = ndi_1 BMI__kg_M2 ASCVD_10_yr / dist=normal
      link=identity stdcoef solution;
      random int / subject=geoid type=un;
      run;
```
